# Evaluation of systemic inflammatory response syndrome criteria in horses with colic associated with acute gastrointestinal disease

**DOI:** 10.3389/fvets.2026.1822426

**Published:** 2026-06-04

**Authors:** V. Biondi, M. Pugliese, P. Gambadauro, C. Faraci, D. A. Sicuso, G. Bruschetta, A. Passantino, G. Catone, C. Vullo

**Affiliations:** 1 Department of Veterinary Sciences, University of Messina, Messina, Italy; 2 Department of Chemical, Biological, Pharmaceutical, and Environmental Sciences, University of Messina, Messina, Italy

**Keywords:** clinical and laboratory variables, colic syndrome, horses, outcome, systemic inflammatory response syndrome

## Abstract

**Background:**

Systemic inflammatory response syndrome (SIRS) is a severe clinical condition commonly observed in horses with acute gastrointestinal disease associated with colic and is linked to increased risk of organ dysfunction and mortality. Early identification of SIRS and reliable prognostic indicators are essential to improve clinical management. This study aimed to assess the occurrence of SIRS in adult horses with colic, evaluate its association with outcome, and investigate the prognostic value of selected clinicopathological variables.

**Materials and methods:**

A total of 41 adult horses admitted for acute gastrointestinal disease were included. Clinical, hematological, and biochemical data were collected at admission. A SIRS score (0–6) was calculated based on temperature, heart rate, respiratory rate, white blood cell count, blood lactate concentration, and mucous membrane characteristics. Horses were classified as SIRS-positive (≥2 criteria) or SIRS-negative. Outcomes were recorded as survivors (SUR) or non-survivors (NSUR). Statistical analyses included group comparisons and linear regression to evaluate associations between variables and survival.

**Results:**

SIRS was identified in 58.5% (24/41) of horses. Mortality was significantly higher in the SIRS-positive group (50%) compared to the SIRS-negative group (11.7%). SIRS-positive horses showed increased heart and respiratory rates and significant eosinopenia. Among SIRS-positive horses, non-survivors had significantly higher blood lactate concentrations than survivors (*p* = 0.04). Linear regression analysis demonstrated a significant inverse relationship between eosinophil count and survival time, indicating that higher eosinophil levels were associated with shorter survival.

**Conclusion:**

SIRS is associated with increased mortality in horses with acute gastrointestinal disease. Blood lactate concentration and eosinophil count emerged as relevant prognostic indicators. The combined use of SIRS scoring and selected laboratory parameters may support early risk stratification and clinical decision-making in equine emergency medicine. Further prospective studies are needed to confirm these findings.

## 1 Introduction

Systemic inflammatory response syndrome (SIRS) is a clinical condition characterized by a systemic inflammatory response induced by infection, trauma, or other conditions that cause an imbalance in the immune system. This condition leads to a pathophysiological reaction involving the uncontrolled release of inflammatory mediators capable of harming various tissues and organs, which consequently causes additional organ system dysfunction and an increased risk of mortality during hospitalization (1).

External injuries stimulate horses’ immunological system more than others; consequently, horses are more predisposed to SIRS due to trauma, infections, and to acute gastrointestinal diseases associated with colic syndrome (2). In adult horses, several gastrointestinal diseases such as colic syndromes, colitis, esophageal obstructions, intra-abdominal abscesses, peritonitis, and neoplasms can trigger SIRS (2). The risk of SIRS increases during ischemic injury in most cases of colic syndrome due to the consequent loss of mucosal integrity that allows bacterial entry and translocation of bacterial endotoxins into the bloodstream (3).

The relationship between the inflammatory response and the mechanisms that regulate inflammation determines the occurrence of SIRS. Underlying the pathophysiological mechanism of SIRS, the receptors of innate immunity (PRRs, Pattern Recognition Receptors), which are found mainly in circulating monocytes, play a fundamental role. Their function is to recognize and bind bacterial endotoxins (LPS, Lipopolysaccharide), which are the main components of the wall of Gram-negative bacteria. Indeed, it is the activation of PRRs that predisposes to the increase of cytokines and other inflammatory mediators that are responsible for most clinical signs observed in equine patients (4). Endotoxemia and SIRS can occur by promoting the transport of bacterial endotoxins into the general circulation. This leads to systemic inflammation, initially characterized by a local systemic inflammatory response, which, if not well controlled, evolves into a massive inflammatory response. The local systemic inflammatory response aims to limit organic injuries through cellular recruitment and activation of neutrophils, basophils, and eosinophils, resulting in vasodilation, increased permeability, and endothelial damage.

Horses, compared to other species, have a greater tendency to develop colic due to the anatomical and functional characteristics of their digestive system (5). Colic syndrome, before evolving into SIRS, manifests itself as endotoxemia, which can be caused by bacterial infections sustained by *Neorickettsia risticii, Clostridium perfrigens, Clostridium difficile,* and Salmonella spp., as well as inflammatory bowel disease and strangulation (6). The endotoxic picture, however, can occur even in the absence of infectious agents, precisely because when the gastrointestinal mucosa is compromised, endotoxins from the normal bacterial flora may migrate into the bloodstream (6).

To overcome the difficulties of diagnosis, a list of criteria, including clinical and laboratory data to objectify the presence of SIRS, has been defined (7). It is an easy-to-use and inexpensive method that confirms or excludes the presence of a systemic inflammatory response without attributing any prognostic value.

The list of criteria to confirm the presence of SIRS was devised for the first time in 2005 by Corley et al. (8), applying to horses the international guidelines established by the American College of Chest Physicians/Society of Critical Care Medicine Consensus Conference (ACCP/SCCM) held in 1991. Body temperature, heart rate, respiratory rate, and blood leucocyte count were considered criteria useful to identify the SIRS occurrence. A patient is defined as SIRS positive if two out of four altered parameters are present (9).

In 2017, Roy et al. (2) defined the presence of SIRS in a population of adult equines admitted to an emergency based on meeting at least two of the following parameters:

(i) Rectal temperature >38.5 °C or <37 °C
(ii) Heart rate >52 bpm
(iii) Respiratory rate >20 rrm
(iv) WBC > 12.5 K/μL or <5.0 K/μL.

Other studies have suggested adding two more criteria, lowly expensive and easy to assess, to be considered in the diagnosis of SIRS, such as the assessment of mucosal color and serum lactate concentration (2, 10). These parameters provide an additional assessment for the presence of SIRS in adult horses, considering physiological changes that can be identified quickly and accurately, and represent, with other variables, a good model to estimate the prognosis.

We hypothesize that the occurrence of SIRS in horses with colic may influence clinical outcome and that additional clinicopathological parameters could contribute to the assessment of disease severity. Therefore, this study aimed to evaluate the presence of SIRS in a population of adult horses with acute gastrointestinal disease and to investigate its association with outcome. In addition, given the use of an extended scoring system, the study sought to explore its potential clinical utility and to compare its performance with that of the traditional SIRS definition.

## 2 Materials and methods

### 2.1 Animals and clinical procedures

The study population consisted of horses (aged ≥1 year) admitted to an equine referral hospital between May 2021 and April 2023 with a diagnosis of acute gastrointestinal disease associated with colic syndrome.

Clinical data recorded on medical records, such as signalment, physical examination results, final diagnosis, and outcome, were prospectively collected and retrospectively analyzed. All owners signed informed consent for the use of clinical and diagnostic data. All procedures were carried out in accordance with the standards set by Italian and European rules on animal welfare and good veterinary practice. Upon admission, each horse’s clinical history and general condition were recorded, followed by a comprehensive physical, hematological, and biochemical evaluation, including measurement of blood lactate concentration. The physical examination included assessment of rectal temperature, respiratory rate, intestinal sounds, presence of spontaneous gastric reflux, digital pulse quality, heart rate, capillary refill time, and mucous membrane color. Horses with colic commonly exhibited clinical signs such as pawing, rolling, flank watching, sweating, and restlessness. Blood samples were collected from the jugular vein and stored in three tubes. A test tube containing potassium ethylenediaminetetraacetic acid (K_2_EDTA) was used to perform a complete blood cell count (CBC) with a semiautomatic electronic blood cell counter validated for horses (Procyte Dx, Idexx, Milan, Italy). Also, the leukocyte differential count, including eosinophil cell count, was carried out manually on blood smears. Peripheral blood smears were air-dried and stained using a Romanowsky-type stain (Wright–Giemsa). Slides were examined under oil immersion (1,000×) in the monolayer region using a systematic scanning pattern. Eosinophils were identified based on their bilobed nucleus and abundant cytoplasm containing prominent eosinophilic granules and included in a 100-cell leukocyte differential count. Results were expressed as a percentage of total leukocytes; absolute eosinophil counts were calculated based on total leukocyte counts.

To obtain serum samples for biochemical tests evaluation, including electrolyte evaluations, a sample collected into a test tube with a cloth activator was centrifuged at 3,000 rpm for 10 min (within 30 min). Glucose, blood urea nitrogen (BUN), creatinine, total proteins, albumin, gamma-glutamyl transferase.

GGT was assayed using a chemistry analyzer (Catalyst Dx, Idexx, Milan, Italy).

A third rate was drawn into a tube containing lithium-heparin as the anticoagulant to assay blood lactate levels (Catalyst Dx, Idexx, Milan, Italy).

A scoring system was applied to categorize these clinical and laboratory findings suggestive of SIRS. The identification of SIRS was performed and assessed in the presence of at least two of the following four parameters, recorded during the clinical examination at the time of admission to the emergency room:

(i) Hypothermia/hyperthermia (below 37 °C or above 38.5 °C)
(ii) Tachycardia (>52 bpm)
(iii) Tachypnoea (>20 rrm)
(iv) Abnormal white blood cell count (>12,500 cells/μL or <5,000 cells/μL and 10% neutrophils) (2).

Moreover, the blood lactate concentration (normal blood lactate: ≤2.06 mmol/L; abnormal >2.06 mmol/L) and color of the mucous membranes were evaluated as clinical variables. Mucous membranes were defined as abnormal if they appeared pale (white), injected (red), cyanotic (purplish), or presented hemorrhagic petechiae, all indicators of compromised peripheral perfusion. They were measured as clinical parameters consistent with SIRS and contributed to clinical scores (2). Each variable was scored from 0 to 1 according to the alteration of the clinical sign considered (Table 1). Therefore, a point-scale SIRS score ranged between 0 and 6 (2), implying that scores of 0–1 were negative and 2–6 were positive.

**Table 1:** Clinical scoring system used for horses affected with acute gastrointestinal diseases associated with colic syndrome.

Variables (x)	0*	1*
Body temperature	37.0 °C < x < 38.5 °C	x < 37.0 °C or x > 39.7 °C
Pulse rate	x < 52 bpm	x > 52 bpm
Respiratory rate	x < 20 rrm	x > 20 rrm
Abnormal white blood cell count	5,000 cells/μL <x < 12,500 cells/μL	5,000 cells/μL or x < x > 12,000 cells/μL > neutrophils 10%
Blood lactate concentration	x ≤ 2.06 mmol/L	x > 2.06 mmol/L
Mucous membranes	Normal	Abnormal

*Score.

Based on the presence of SIRS, horses were divided into two groups: Pos Group (Positive for SIRS, with at least 2 SIRS criteria) and Neg Group (Negative for SIRS, with 0–1 SIRS criteria). Within the Pos Group, horses were further classified into Survivors (SUR) and Non-survivors (NSUR) at discharge. The NSUR group included horses that died spontaneously or were euthanized due to a poor clinical prognosis. Horses euthanized solely for financial constraints were excluded from the study.

### 2.2 Statistical analysis

Statistical analysis was performed using commercially available software (Statistical Package for the Social Sciences 13.0 for Windows; SPSS Inc., Chicago, IL, USA). Categorical variables [race, gender (male/female)] were expressed in frequencies. The normality of the data was assessed using the Kolmogorov–Smirnov test. Numerical variables such as age and SIRS score were expressed as mean and standard deviation. The Mann–Whitney test was used to compare numerical variables. Fisher’s exact test was applied to compare the survival of patients. The overall mortality rate was also calculated. Additionally, multiple linear regression analysis was conducted to assess the association between selected clinicopathological variables (heart rate, respiratory rate, eosinophil count, and lactate concentration) and horses’ outcome. Statistical significance was set at a *p*-value of ≤0.05.

## 3 Results

**Figure 1:**
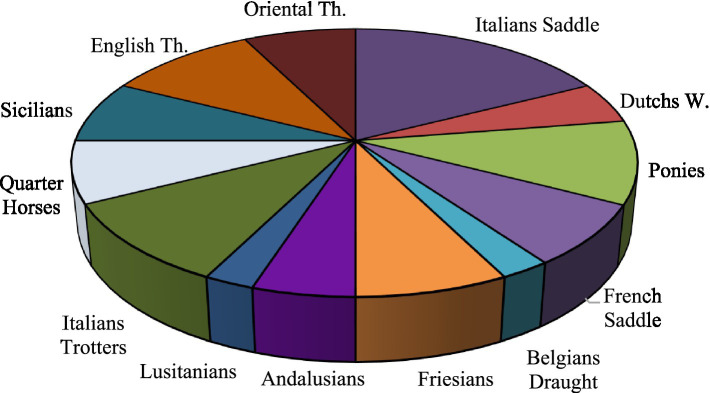
Breed distribution of horses affected by acute gastrointestinal disease included in the study.

A total of 41 horses was included, consisting of 18 females, 14 geldings and 9 males. The mean age was 7.9 years (median 6 years; range 2–20 years; IQR 4–12.2 years). The horses belonged to different breeds (*n* = 7/41 Italian Saddles [17.1%], *n* = 2/41 Dutch Warmbloods [4.9%], *n* = 4/41 Ponies [9.8%], *n* = 3/41 French Saddles [7.3%], *n* = 1/41 Belgian Drafts [2.4%], *n* = 3/41 Friesian [7.3%], *n* = 2/41 Andalusians [4.9%], *n* = 1/41 Lusitanos [2.4%], *n* = 4/41 Italian Trotters [9.8%], *n* = 3/41 Quarter horses [7.3%], *n* = 3/41 Sicilians [7.3%], *n* = 4/41 English Thoroughbreds [9.8%], and *n* = 3/41 Oriental Thoroughbreds [7.3%]) (Figure 1).

Based on the scoring criteria, 24 horses were identified as SIRS-positive and assigned to the Pos Group, while 17 horses were classified as SIRS-negative and included in the Neg Group.

Concerning the diagnosis (classified as either non-strangulating or strangulating colic), 32 horses were diagnosed with non-strangulating colic (17/24; 70.8%) in the Pos Group and 15/17 (88.2%) in the Neg Group. Diagnosis was confirmed in all cases via exploratory celiotomy or necropsy (10, 11). Concerning the diagnosis (classified as either non-strangulating or strangulating colic), 32 horses were diagnosed with non-strangulating colic (17/24; 70.8%) in the Pos Group and 15/17 (88.2%) in the Neg Group, and 7 horses were diagnosed with strangulated colic in the Pos group (7/24; 29.2%) and 2/17 (11.8%) in the Neg group. Diagnosis was confirmed in all cases via exploratory celiotomy or necropsy (10, 11). Constipation was recorded in 29/41 cases (70.7%), including 15/24 (62.5%) in the Pos group and 14/17 (82.4%) in the Neg group. Bloating-associated colic occurred in 3/41 cases (7.3%), with 2/24 (8.3%) in the Pos group and 1/17 (5.9%) in the Neg group.

Strangulating colic was observed in 9/41 cases (22.0%) and was attributable to torsion, intussusception, and/or mesenteric hernia; this included 7/24 (29.2%) cases in the Pos group and 2/17 (11.8%) in the Neg group. All such cases required total colectomy with end-to-end anastomosis.

Concerning the horses’ ability to survive, the clinical diagnoses had a substantial impact (*p* = 0.001, *p <* 0.001). Our study found that 1% of the horses identified with incarcerated inguinal hernia survived, while 22% did not. Horses suffering from meteorism exhibited a survival rate of 4%, whereas 17% did not survive. Patients diagnosed with first-degree stomach distension, intestinal strangulation, and peritonitis had a survival rate of 5%, while the non-survival rate was 11%, respectively.

**Figure 2:**
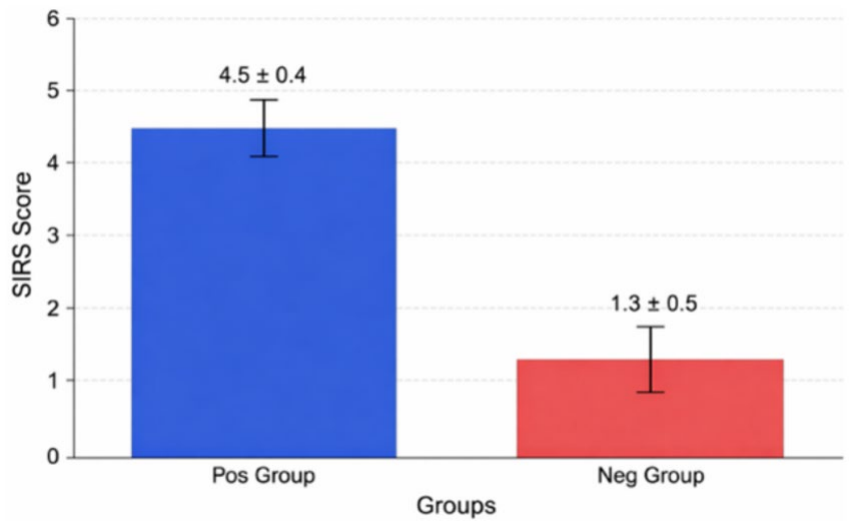
Comparison of SIRS score between positive and negative groups (Pos group and Neg group).

The mean SIRS score was significantly higher (*p* = 0.01) in the Pos Group (4.5 ± 0.4) than the Neg Group (Figure 2).

**Figure 3:**
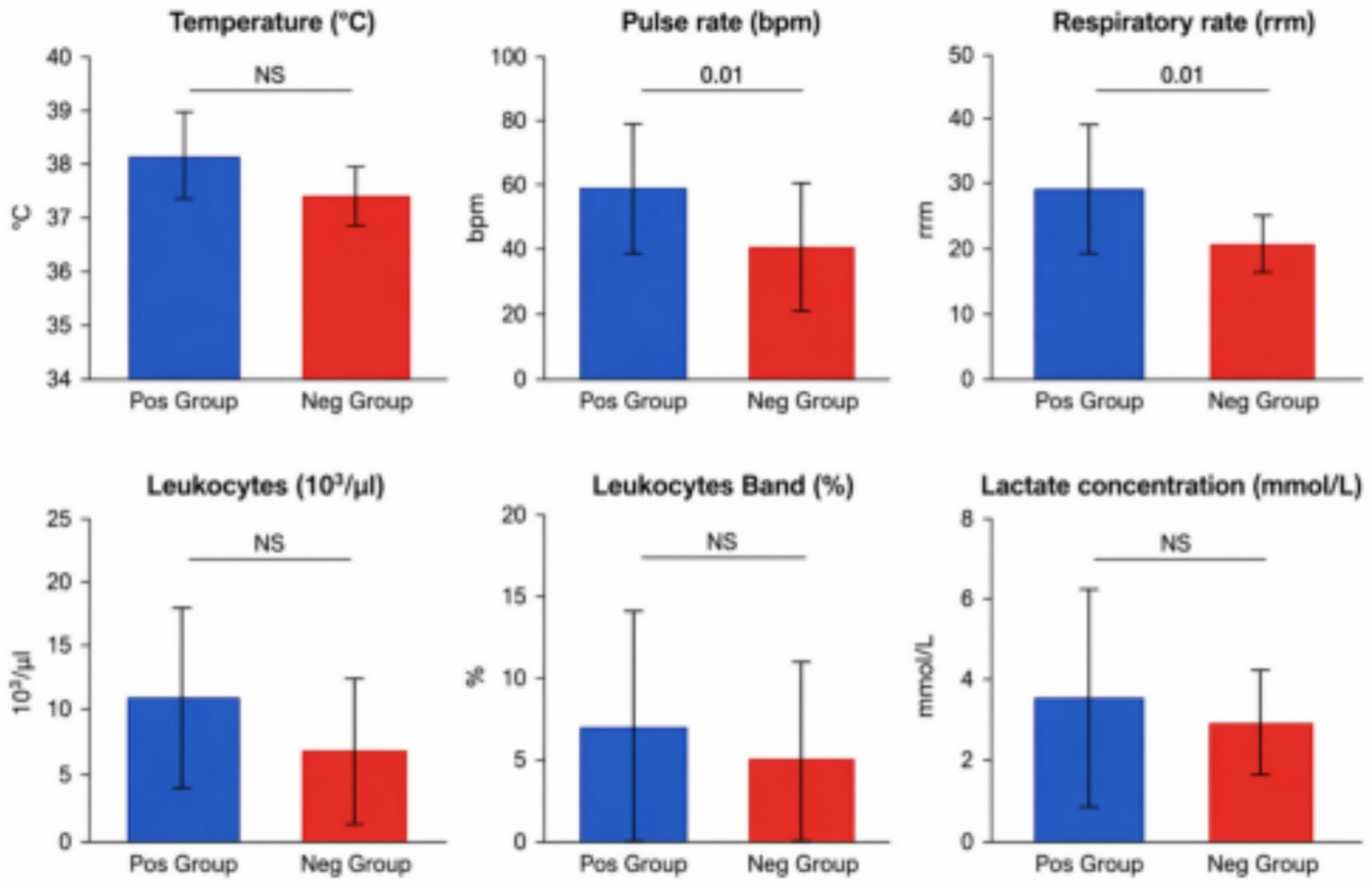
Clinical variables identifying SIRS recorded in the two groups (Pos and Neg), and their significance (*p*-value). NS, not significant (*p* ≥ 0.05).

Horses in the Pos Group showed significantly higher values of pulse rate (*p* = 0.01) and respiratory rate (*p* < 0.01) than the Neg Group (Figure 3).

Regarding mucous membrane evaluation, abnormal findings were recorded in 6/24 (25%) horses in the Pos Group and 3/17 (17.6%) horses in the Neg group. However, no statistically significant difference was observed between the two groups for this parameter.

Regarding other variables included in the clinical score, such as body temperature and white blood cell (WBC) count, no significant differences were found between the two groups. Data regarding body temperature, pulse rate, respiratory rate, total WBC count, band leukocytes, and lactate blood concentrations for both groups are summarized in Figure 3.

**Figure 4:**
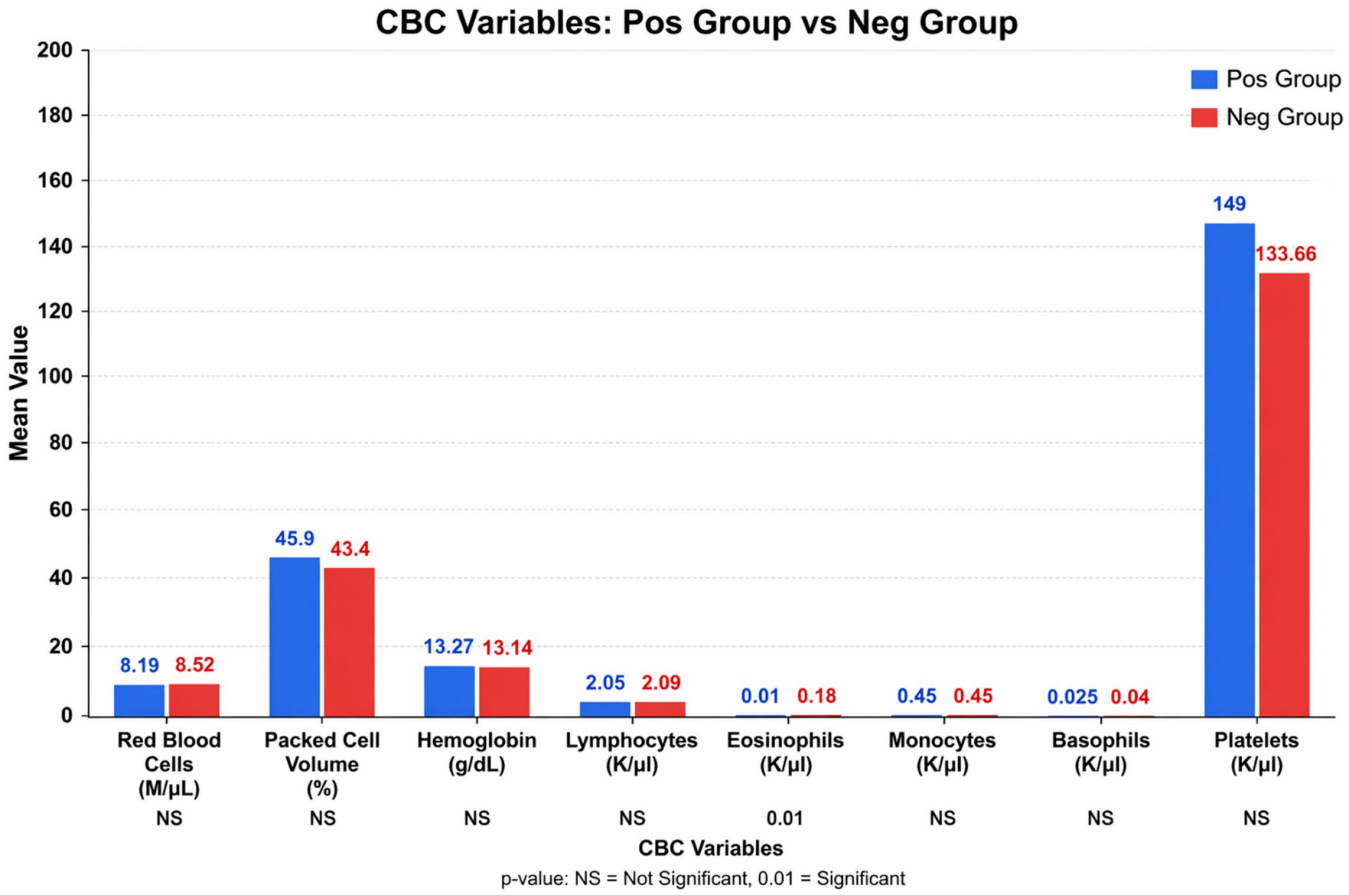
Variables included in the blood count cells recorded in horses enrolled and affected with acute gastrointestinal diseases associated with colic syndrome.

The parameters referable to CBC were not statistically significant, except for the eosinophil count, which was significantly lower (*p* = 0.04) in the Pos Group compared to the Neg Group (0.01 ± 0.04 vs. 0.18 ± 0.17) (Figure 4).

**Figure 5:**
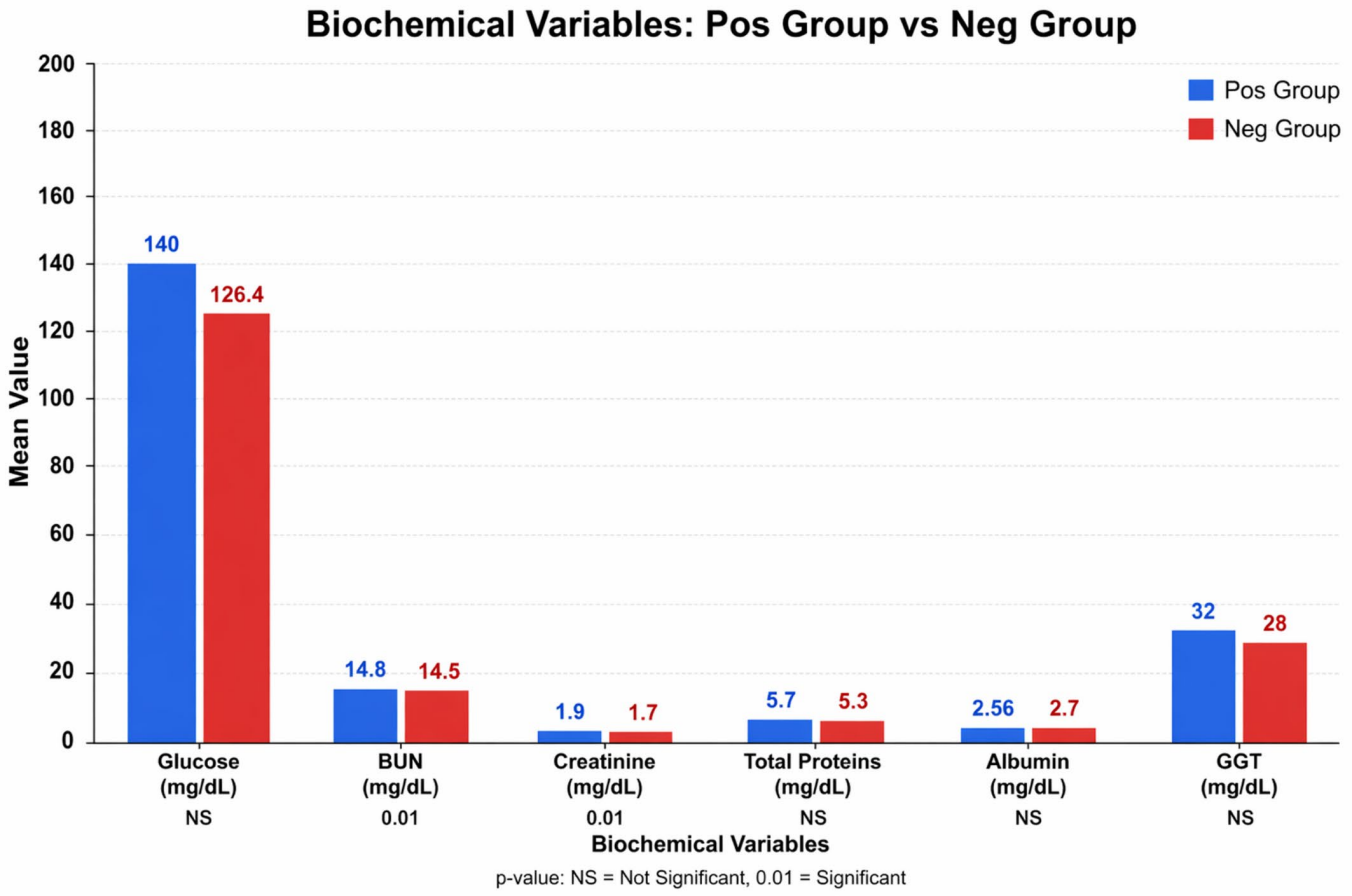
Biochemical variables recorded in horses enrolled and affected with acute gastrointestinal diseases associated with colic syndrome.

When comparing the two groups, no statistically significant differences were observed among the biochemical parameters, except for BUN and creatinine concentrations (Figure 5). Both parameters were significantly higher in the Pos Group compared to the Neg Group (*p* = 0.01).

**Figure 6:**
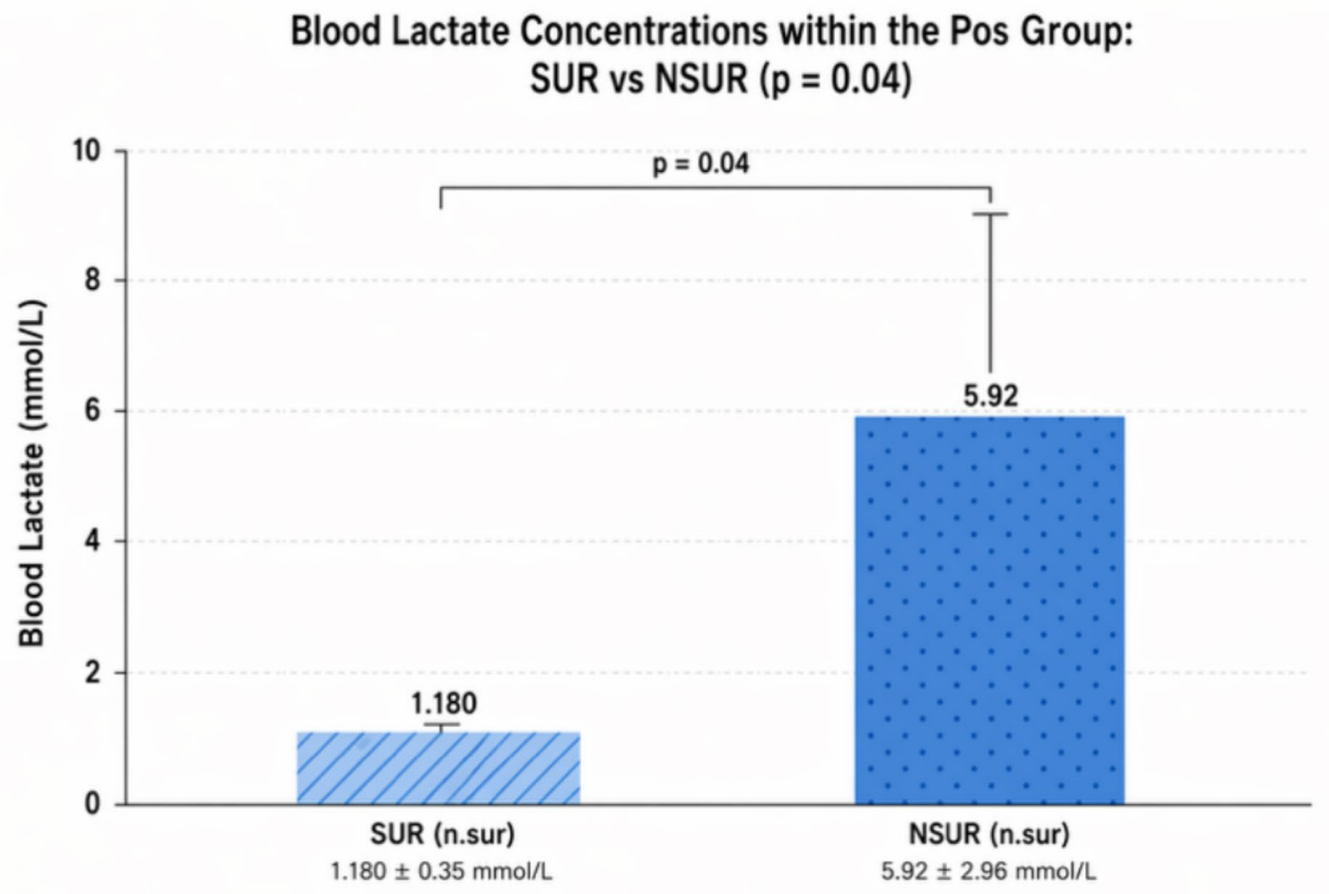
Blood lactate concentration in the POS group. The figure shows a comparison between horses with acute gastrointestinal diseases associated with colic syndrome, categorized as survivors (SUR) and non-survivors (NSUR).

Within the Pos Group, a comparison between SUR and NSUR revealed that blood lactate concentrations were significantly higher in the NSUR group than in the SUR group (5.92 ± 2.96 mmol vs 1.180 ± 0.35 mmoL/L; *p* = 0.04) (Figure 6).

The overall mortality rate for the entire study population was 34.1% (14/41). The mortality rate for the Pos Group was 50% (12/24), and for the Neg Group, 11.7% (2/17). Horses that died in the Neg Group had a clinical score of 1, while those that died in the Pos Group had clinical scores between 5 and 6. Regarding the clinical management of the Pos Group, seven horses (29%) were euthanized at admission or shortly thereafter due to a grave prognosis, and seven horses (29%) underwent emergency surgery, of which five died during or after the surgery. In the Neg Group, the two non-surviving horses died during surgery.

**Figure 7:**
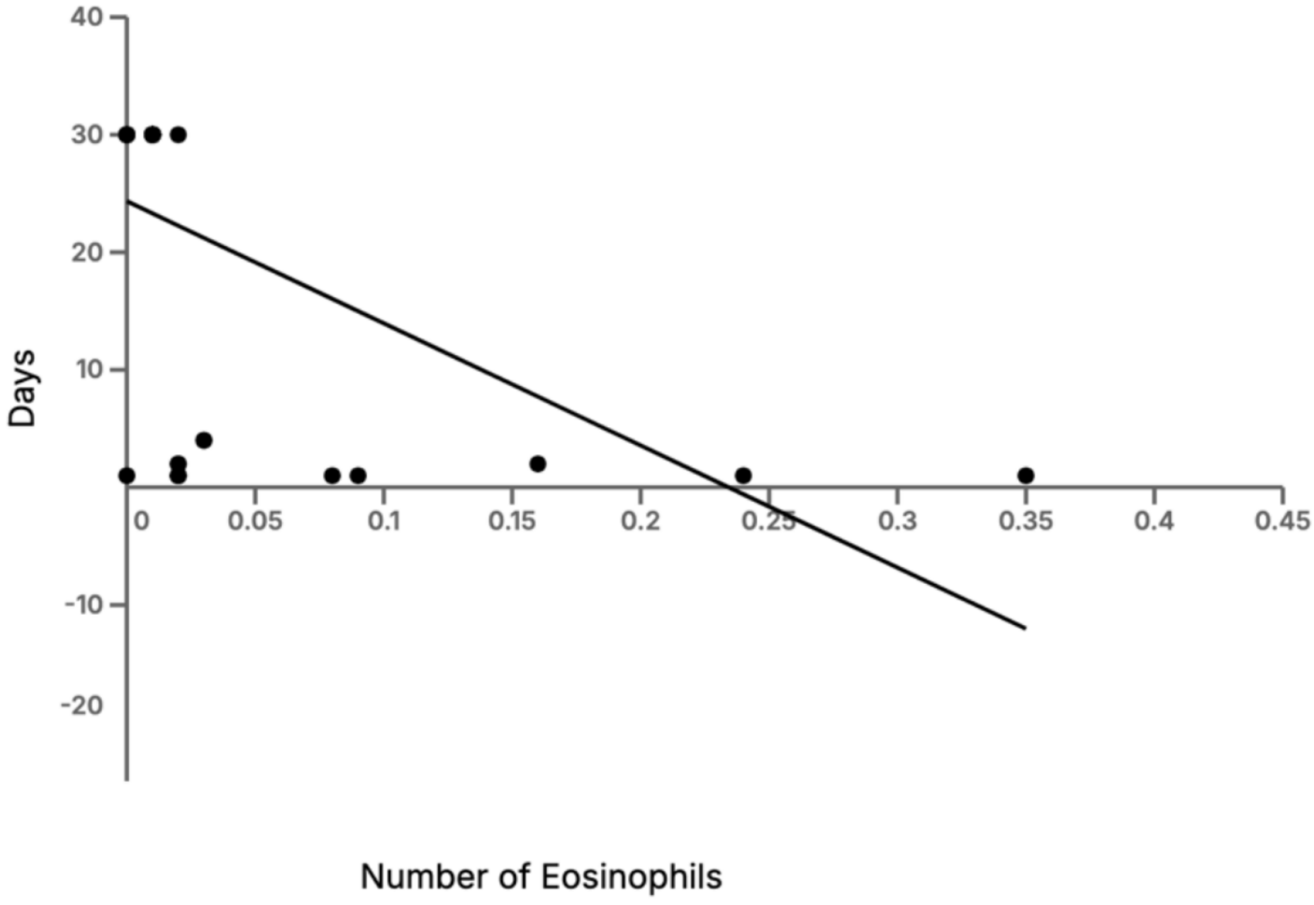
The linear regression analysis suggests that elevated eosinophil levels are associated with reduced survival.

The linear regression analysis demonstrated a significant inverse relationship between eosinophil count and survival time (Figure 7). Specifically, eosinophil levels were negatively associated with days of survival (slope = −103.9 ± 26.7; 95% CI: −157.9 to −49.9; *p* = 0.0004), indicating that higher eosinophil counts correspond to shorter survival. The model showed a moderate goodness of fit (*R*^2^ = 0.2795), suggesting that approximately 28% of the variability in survival time is explained by eosinophil levels alone. The regression equation (Y = −103.9X + 24.34) further supports the observed inverse trend. Despite the relatively modest *R*^2^ value, the association remained statistically robust, as confirmed by the significant *F*-test (*F* (1,39) = 15.13, *p* = 0.0004). These findings indicate that eosinophil count is a significant predictor of survival, although additional factors are likely to contribute to the observed variability.

**Figure 8:**
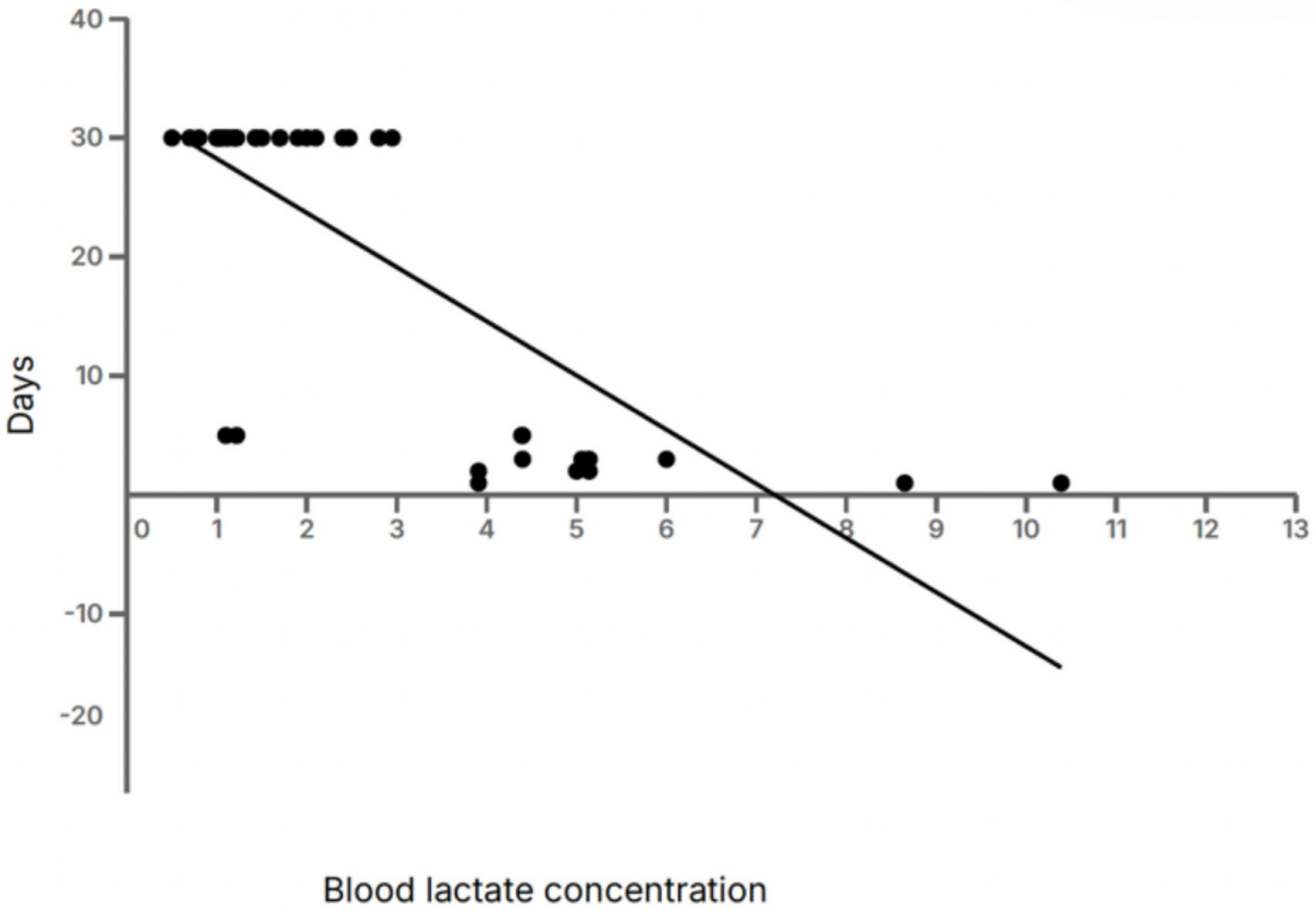
The linear regression analysis suggests that elevated blood lactate levels are associated with shorter survival times.

Linear regression analysis revealed a significant negative association between blood lactate concentration and survival days (slope = −4.553 ± 0.599; 95% CI: −5.765 to −3.342; *R*^2^ = 0.597; *p* < 0.0001). Higher blood lactate concentrations were associated with shorter survival times (Figure 8).

## 4 Discussion

SIRS management remains one of the most complex challenges in equine medicine. In these patients, SIRS is frequently associated with gastrointestinal disorders, musculoskeletal and skin problems (massive wounds, burns, and lacerations), joint disorders (septic arthritis, laminitis, tendonitis, and fractures), or postnatal conditions such as septic metritis. Diseases affecting the liver, respiratory system, urogenital system, and immune system are generally considered lower-risk triggers. Within this framework, gastrointestinal diseases, such as colic syndromes, colitis, esophageal obstructions, intra-abdominal abscesses, peritonitis, and neoplasms, are recognized as the main cause of SIRS (2).

The pathogenic mechanisms leading to SIRS in horses are mainly due to the onset of endotoxemia. The systemic inflammatory response follows an inefficient immune response, resulting in the amplified activation of inflammatory cells and the uncontrolled release of soluble pro-inflammatory mediators (12, 13). This results in interstitial and tissue damage, exacerbated by oxygen-free radicals, localized in the proteases released by the polymorphonuclear cells (neutrophils, eosinophils, and basophils). In addition, a decrease in antithrombin III levels, resulting in the formation of microthrombi and consequently, in an alteration of coagulation and microcirculation, can appear. Concurrent vasodilation causes a loss of muscle tone, resulting in a drop in intravascular pressure, aggravated by an imbalance of mediators regulating vascular smooth muscles (14). These mechanisms cause a reduction in tissue oxygen supply, leading to an activation of anaerobic metabolism and an increased blood lactate concentration. Because of insufficient oxygen supply, multi-organ dysfunction affecting the kidneys, heart, and brain occurs. In any case, during the massive systemic inflammatory response, an excessive immunosuppression, known as ‘anti-inflammatory response syndrome, may appear (15, 16).

The overactivity of the anti-inflammatory response predisposes the patient to a stage of immunosuppression, making them more susceptible to secondary bacterial infections, with a very high mortality rate due to the ineffectiveness of the immune system (12). In addition, as high levels of IL-10 and TGF occur, there is a decrease in the expression of MCH-II, a decrease in the release of pro-inflammatory cytokines and free radicals, and an inhibition of lymphocyte proliferation (17, 18). Decreased cytokine release leads to an inhibition of macrophage activation, causing a communicative disruption between T- and B-cells, resulting in reduced antibody synthesis. Finally, a local dysfunction of neutrophils, basophils, and eosinophils ensues (12).

A resulting endotoxemia can be caused by sustained bacterial infections, but also in the absence of infectious agents, precisely because of the compromise of the gastrointestinal mucosa, as in the case of inflammatory bowel disease or strangulation, in which endotoxins from the normal bacterial flora can migrate into the blood circulation (6).

The syndrome presents non-specific clinical signs such as hyperthermia or hypothermia, tachycardia, tachypnoea, cyanotic mucous membranes with hemorrhagic petechiae or with a toxic ring, and increased capillary refill time (19).

Hyperthermia or hypothermia appears as an alteration of the thermoregulatory center, while hypotension (systolic pressure <90 mmHg or basal pressure of 40 mmHg) and endothelial damage are responsible for compensatory tachycardia (20). Tachypnoea is a consequence of respiratory difficulty caused by edema and increased anaerobic metabolism (metabolic acidosis) (20). Patients may present with anorexia, symptoms of abdominal pain in the case of colic syndrome, and an altered mental state (depression of the sensorium) (21), the latter due to cerebral edema caused by increased vascular permeability (20). Indeed, the assessment of mucous membrane color has considerable diagnostic importance, as the observable changes are often an expression of systemic pathologies. The examination in this case is dedicated to the explorable mucous membranes, such as the conjunctival and oral mucosa.

Clinical signs are often associated with alterations in the hematological, biochemical, and coagulative tests. The CBC reveals an alteration in the leukogram pattern, such as leukocytosis or leukopenia, neutrophilia or neutropenia, or neutrophil left shift, which are related to the inflammatory process. An increased packed cell volume due to the fluid extravasation by increased vascular permeability can be observed (2).

The assessment of lactate blood concentration caused by the drop in pressure also has considerable diagnostic importance during colic (2, 22).

The difficulty of diagnosing SIRS is among the main factors influencing the poor prognosis, and it is, therefore, essential to optimize the protocols for identifying the syndrome. To do this, the SIRS score was developed, a collection of clinical and laboratory data that confirms or excludes the presence of an inflammatory response (7).

According to the results obtained from this study, the mortality rate of SIRS-positive horses was higher than that of SIRS-negative horses, providing evidence that acute gastrointestinal diseases associated with SIRS have a greater likelihood of an unfavorable prognosis, as published previously by other authors (2, 5, 23–25). This study showed that SIRS is clinically relevant in horses, being a potentially fatal complication of colic syndrome in adult horses. Its correlation with endotoxic shock and septic shock predisposes to a high risk of multi-organ dysfunction and consequently increased mortality.

A percentage of horses died during or after the surgery, suggesting that the onset of SIRS with other risk factors can worsen the prognosis of patients.

The present study also investigated prognostic variables related to SIRS in a population of adult horses suffering from acute gastrointestinal disease. The results showed a higher mortality rate in SIRS-positive horses (Pos Group) than in SIRS-negative horses (Neg Group). Furthermore, horses in the Pos group had significant, and a significant increase in pulse rate and respiratory rate, compared to the Neg group. In addition, within the SIRS-positive group, significant blood lactate levels were evident in the horses that died (NSUR), compared to those that remained alive (SUR).

The present analysis reveals a significant inverse relationship between eosinophil count and survival time, indicating that higher eosinophil levels may be associated with less favorable clinical outcomes. From a pathophysiological standpoint, eosinophils are multifunctional leukocytes involved in both inflammatory and immune processes; their elevation may therefore reflect a state of systemic activation or dysregulation.

In various clinical settings, eosinophilia has been linked to chronic inflammation, tissue injury, and immune-mediated mechanisms, all of which can contribute to disease progression and reduced survival (26–28). Eosinophils can release cytotoxic granule proteins, cytokines, and reactive oxygen species, potentially amplifying tissue damage and organ dysfunction—mechanisms that may partly account for the negative association observed in this study.

At the same time, increased eosinophil counts may represent a marker of disease severity rather than a direct causal factor. Eosinophilia has been described in association with parasitic infections, allergic disorders, neoplasia, and inflammatory syndromes, conditions that can independently worsen prognosis. Thus, the observed relationship with survival may reflect the overall burden of underlying disease processes.

In human medicine, eosinopenia has long been considered an indicator of sepsis and systemic inflammatory response syndrome (SIRS), particularly in critically ill patients and those presenting with abdominal pain (29, 30). More recently, its potential utility has also been explored in veterinary medicine as a rapid marker to identify patients requiring intensive care (31). In horses, however, eosinophil counts are physiologically low and may decrease further in response to activation of the hypothalamic–pituitary–adrenal axis during stress and critical illness (32). In this context, eosinopenia is more likely to reflect cortisol-mediated stress responses rather than a specific marker of systemic inflammation. Indeed, although catecholamines such as epinephrine may contribute to leukocyte redistribution, an epinephrine-mediated stress leukogram would generally also be associated with neutrophilia and lymphocytosis, a pattern not consistently observed in critically ill horses (32). Conversely, previous studies have demonstrated a correlation between cortisol concentrations and eosinophil counts in horses, supporting the hypothesis that glucocorticoid-mediated mechanisms play a predominant role in eosinophil reduction during acute inflammatory and critical conditions (32–35).

Overall, these findings are consistent with previous evidence supporting a prognostic role for inflammatory biomarkers, although the direction and strength of the association may vary depending on the disease context and study population. It is also important to note that the use of linear regression represents a simplified analytical approach; future studies incorporating time-to-event methods, such as Cox proportional hazards models, would allow for a more comprehensive assessment of survival dynamics.

A study published by Martin-Cuervo et al. (31) focused on the evaluation of eosinopenia as a marker of SIRS in severely ill horses. Comparing the results of sick horses with a control group of healthy horses showed how eosinophilic count can help to discriminate between healthy and SIRS horses. Also, it was reported that non-surviving horses showed lower eosinophil counts than survivors. From reading these results, the study team considered eosinopenia as a possible accurate variable in differentiating SIRS-infected horses from unaffected horses. Similarly, our data showed that horses in the SIRS group had significant eosinopenia compared with those in the SIRS-negative group. This suggests its validity as a discriminator of SIRS, as it clearly indicates the presence of an acute inflammatory process and is therefore a negative prognostic factor.

The present analysis demonstrates a significant inverse association between blood lactate concentration and time, as evidenced by the linear regression model. This finding indicates a progressive reduction in lactate levels over the observed period, suggesting an effective physiological or clinical process underlying lactate clearance. In a healthy horse, lactatemia does not exceed 2.00 mmol/L in blood and 0.50–0.99 mmol/L in plasma (22; 27). During gastrointestinal colic, when intestinal ischemia occurs, the intestinal permeability is altered and anaerobic cellular metabolism is established, leading to the production of lactate and its passage into the general circulation stream (22). There are many causes of increased lactate concentrations in blood and other biological fluids, but the most common and important causes are alterations in tissue perfusion and hypoxia. Also, the increase in plasma lactate concentration can occur in critically ill patients suffering from diseases where oxygen transport to the tissues is normal. Indeed, when lactate production from hypoxic tissues exceeds the rate of elimination through the kidneys and liver, its concentration increases in the blood (36–38). In our study, all SIRS-positive horses presented a significant increase in lactate rate, even if not statistically significant. This is explained by general tissue suffering caused by SIRS during colic. Notably, non-survivor horses presented lactate concentrations higher than those of survivors tested during hospitalization. This data could represent a negative prognostic aspect in horses, suggesting a poor compensation for hypoxic tissue capacity in any patients affected by colic syndrome.

Several previous studies have evaluated the consistency of certain parameters in colic horses and their correlation with outcome. Although results vary across studies, the assessment of cardiovascular status, including heart rate, packed cell volume, and abnormal mucous membrane color, was usually correlated with outcome (36–39). Similarly, the same factors, including blood creatinine concentration, band neutrophils, and base excess, are also reported to be associated with outcomes in horses with colitis (40). Blood lactate concentration is described as a parameter to correlate with prognosis during gastrointestinal diseases (41), in equine emergencies (42) and in SIRS horses (2). The results reported in this study support data previously published, highlighting the significance of assessing the cardiovascular status of horses in SIRS evaluation as a complication of gastrointestinal emergencies. Although the heterogeneity of clinical cases is related to the diagnosis, chronicity, and comorbidities, it is possible to consider that the presence of SIRS is correlated with a more negative outcome. In these cases, performing an early diagnosis and, consequently, an adequate prompt treatment could be the primary objective in managing a patient affected with SIRS, to achieve successful and effective therapy (12), (40).

Conversely, the no-SIRS status does not guarantee a positive outcome if other comorbidities are present (2). Therefore, the continuous patient monitoring as a tool to assess and monitor the horse’s condition results in the primary importance of detecting early the SIRS status.

Limitations of this study include its retrospective design and the relatively small sample size, both of which may have reduced statistical power. In particular, the limited number of cases did not allow for separate statistical analyses based on lesion type or treatment (surgical vs. medical), as further stratification would have resulted in subgroups too small to yield reliable conclusions. In addition, only horses with complete bloodwork were included, potentially introducing a selection bias toward more severe or referred cases. The inclusion of euthanized horses among non-survivors may also have introduced a degree of subjectivity, as decisions can be influenced by clinician judgment and owner preferences.

Another aspect to consider is the heterogeneity of the study population, which comprised horses affected by different types of gastrointestinal disorders. This variability may have influenced the outcomes and limited the ability to conclude specific to individual conditions.

Despite these constraints, the findings provide clinically relevant insights. In particular, the data support an association between the presence of SIRS and increased mortality in adult horses with acute gastrointestinal diseases related to colic.

## 5 Conclusion

SIRS-positive horses showed higher heart and respiratory rates, marked eosinopenia, and increased blood lactate concentrations. Among these variables, hyperlactatemia was significantly associated with non-survival, reinforcing its value as an indicator of tissue hypoperfusion and disease severity. Low eosinophil levels were also linked to reduced survival, suggesting a potential role as a prognostic biomarker, although further studies are needed to clarify the underlying mechanisms and confirm these findings in larger cohorts. Overall, the results highlight a time-dependent decline in lactate levels while underscoring the multifactorial nature of its regulation. Together, these findings support the use of SIRS scoring combined with selected laboratory parameters for early risk stratification and clinical decision-making in equine emergency medicine.

## Data Availability

The raw data supporting the conclusions of this article will be made available by the authors, without undue reservation.

## References

[1] Balk RA. Systemic inflammatory response syndrome (SIRS): where did it come from and is it still relevant today? Virulence. (2014) 5:20–6. doi: 10.4161/viru.27135, 24280933 PMC3916374

[2] Roy MF Kwong GPS. Prognostic value and development of a scoring system in horses with systemic inflammatory response syndrome. J Vet Intern Med. (2017) 31:582–92. doi: 10.1111/jvim.14670, 28207163 PMC5354005

[3] Senior JM Proudman CJ. Plasma endotoxin in horses presented to an equine referral hospital: correlation to selected clinical parameters and outcomes. Equine Vet J. (2011) 43:585–91. doi: 10.1111/j.2042-3306.2010.00328.x, 21496089

[4] Lohmann KL Barton MH. "Systemic inflammatory response syndrome". In: Sellon DC Long MT, editors. Equine Infectious Diseases, 2nd Edn. Amsterdam: Elsevier (2013). p. 119–31.

[5] Farrell A Kersh K Liepman R Dembek KA. Development of a colic scoring system to predict outcome in horses. Front Vet Sci. (2021) 8:697589. doi: 10.3389/fvets.2021.697589, 34692803 PMC8531487

[6] Håkansson A Molin G. Gut microbiota and inflammation. Nutrients. (2011) 3:637–82. doi: 10.3390/nu3060637, 22254115 PMC3257638

[7] Vaala WE House JK. "Neonatal infection". In: Smith BP, editor. Large Animal Internal Medicine, 4th Edn. Amsterdam: Elsevier Mosby (2009). p. 281–91.

[8] Corley KTT Donaldson LL. Arterial lactate concentration, hospital survival, sepsis and SIRS in critically ill neonatal foals. Equine Vet J. (2005) 37:53–9. doi: 10.2746/0425164054406856, 15651735

[9] Bone RC Balk RA Cerra FB Dellinger RP Fein AM Knaus WA . Definitions for sepsis and organ failure and guidelines for the use of innovative therapies in sepsis. Chest. (1992) 101:1644–55. doi: 10.1378/chest.101.6.1644, 1303622

[10] Constable PD Hinchcliff KW Done SH Grünberg W. Veterinary Medicine: A Textbook of the Diseases of Cattle, Horses, Sheep, Pigs and Goats. Peter, editor. 11th ed. St. Louis: Elsevier (2017). p. 1115–28.

[11] Nekouei O Doyle AJ Biermann NM Kaufman JM. Clinical findings, diagnoses, and outcomes of horses presented for colic to a referral hospital in Atlantic Canada (2000–2015). Can Vet J. (2020) 61:281–8. 32165752 PMC7020639

[12] Chakraborty RK Burns B. "Systemic inflammatory response syndrome". In: StatPearls. Ratan Kumar, editor. Treasure Island, FL: StatPearls Publishing (2024)

[13] Matsuda N Hattori Y. Systemic inflammatory response syndrome (SIRS): molecular pathophysiology and gene therapy. J Pharmacol Sci. (2006) 101:189–98. doi: 10.1254/jphs.CRJ06010X 16823257

[14] RR MM DF MA O’Kane CM Silversides JA. Vascular leak in sepsis: physiological basis and potential therapeutic advances. Crit Care. (2024) 28:97. doi: 10.1186/s13054-024-04875-6 38521954 PMC10961003

[15] Ward NS Casserly B Ayala A. The compensatory anti-inflammatory response syndrome (CARS) in critically ill patients. Clin Chest Med. (2008) 29:617–25. doi: 10.1016/j.ccm.2008.06.010, 18954697 PMC2786900

[16] van der Poll T Meijers JCM. Systemic inflammatory response syndrome and compensatory anti-inflammatory response syndrome in sepsis. J Innate Immun. (2010) 2:379–80. doi: 10.1159/000318190, 20606408

[17] Robertson CM Coopersmith CM. The systemic inflammatory response syndrome. Microbes Infect. (2006) 8:1382–9. doi: 10.1016/j.micinf.2005.12.016 16679040

[18] Torre D Tambini R Aristodemo S Gavazzeni G Goglio A Cantamessa C . Anti-inflammatory response of IL-4, IL-10 and TGF-β in patients with systemic inflammatory response syndrome. Mediat Inflamm. (2000) 9:193–5. doi: 10.1080/09629350020002912, 11132778 PMC1781763

[19] Roy MF. Sepsis in adults and foals. Vet Clin North Am Equine Pract. (2004) 20:41–61. doi: 10.1016/j.cveq.2003.12.005 15062458

[20] Blangy-Letheule A Vergnaud A Dupas T Rozec B Lauzier B Leroux AA. Spontaneous sepsis in adult horses: from veterinary to human medicine perspectives. Cells. (2023) 12:1052. doi: 10.3390/cells12071052, 37048125 PMC10093263

[21] Raghavan M Marik PE. Management of sepsis during the early “golden hours”. J Emerg Med. (2006) 31:185–99. doi: 10.1016/j.jemermed.2006.05.008, 17044583

[22] Tennent-Brown BS. Interpreting lactate measurement in critically ill horses: diagnosis, treatment, and prognosis. Compend Contin Educ Vet. (2012) 34:E2. 22271469

[23] López-Martínez MJ Franco-Martínez L Martínez-Subiela S Cerón JJ. Biomarkers of sepsis in pigs, horses and cattle: from acute phase proteins to procalcitonin. Anim Health Res Rev. (2022) 23:82–99. doi: 10.1017/S1466252322000019, 35795920

[24] Zabrecky KA Slovis NM Constable PD Taylor SD. Plasma C-reactive protein and haptoglobin concentrations in critically ill neonatal foals. J Vet Intern Med. (2015) 29:673–7. doi: 10.1111/jvim.12568, 25818221 PMC4895508

[25] Nocera I Bonelli F Vitale V Meucci V Conte G Jose-Cunilleras E . Evaluation of plasmatic procalcitonin in healthy and in systemic inflammatory response syndrome (SIRS) negative or positive colic horses. Animals. (2021) 11:2015. doi: 10.3390/ani11072015, 34359143 PMC8300415

[26] Quirce S Cosío BG España A Blanco R Mullol J Santander C . Management of eosinophil-associated inflammatory diseases: the importance of a multidisciplinary approach. Front Immunol. (2023) 14:1192284. doi: 10.3389/fimmu.2023.1192284 37266434 PMC10229838

[27] Park YM Bochner BS. Eosinophil survival and apoptosis in health and disease. Allergy Asthma Immunol Res. (2010) 2:87–101. doi: 10.4168/aair.2010.2.2.87, 20358022 PMC2846745

[28] Shen ZJ Malter JS. Determinants of eosinophil survival and apoptotic cell death. Apoptosis. (2015) 20:224–34. doi: 10.1007/s10495-014-1072-2, 25563855 PMC5798882

[29] Al Duhailib Z Farooqi M Piticaru J Alhazzani W Nair P. The role of eosinophils in sepsis and acute respiratory distress syndrome: a scoping review. Can J Anaesth. (2021) 68:715–26. doi: 10.1007/s12630-021-01920-8, 33495945 PMC7833890

[30] Hirosawa T Harada Y Morinaga K Takase H Nin M Shimizu T. Eosinopenia as a diagnostic marker of bloodstream infection in a general internal medicine setting: a cohort study. BMC Infect Dis. (2020) 20:85. doi: 10.1186/s12879-020-4814-5, 32000694 PMC6990586

[31] Martín-Cuervo M Gracia-Calvo LA Macías-García B Ezquerra LJ Barrera R. Evaluation of eosinopenia as a SIRS biomarker in critically ill horses. Animals. (2022) 12:3547. doi: 10.3390/ani12243547, 36552467 PMC9774166

[32] Stewart AJ Hackett E Bertin F Towns TJ. Cortisol and adrenocorticotropic hormone concentrations in horses with systemic inflammatory response syndrome. J Vet Intern Med. (2019) 33:2257–66. doi: 10.1111/jvim.15620, 31512777 PMC6766528

[33] Hinchcliff KW Rush BR Farris JW. Evaluation of plasma catecholamine and serum cortisol concentrations in horses with colic. J Am Vet Med Assoc. (2005) 227:276–80. doi: 10.2460/javma.2005.227.276, 16047666

[34] Villalba-Orero M Contreras-Aguilar MD Cerón JJ Fuentes-Romero B Valero-González M Martín-Cuervo M. Association between eosinophil count and cortisol concentrations in equids admitted to the emergency unit with abdominal pain. Animals. (2024) 14:164. doi: 10.3390/ani14010164 38200895 PMC10778409

[35] Brosnahan MM. Eosinophils of the horse: part II—eosinophils in clinical diseases. Equine Vet Educ. (2020) 32:590–602. doi: 10.1111/eve.13262, 40046247

[36] Proudman CJ Edwards GB Barnes J French NP. Modelling long-term survival of horses following surgery for large intestinal disease. Equine Vet J. (2005) 37:366–70. doi: 10.2746/0425164054529328, 16028630

[37] Johnston K Holcombe SJ Hauptman JG. Plasma lactate as a predictor of colonic viability and survival after 360° volvulus of the ascending colon in horses. Vet Surg. (2007) 36:563–7. doi: 10.1111/j.1532-950X.2007.00305.x 17686130

[38] Kaukonen KM Bailey M Pilcher D Cooper DJ Bellomo R. The systemic inflammatory response syndrome criteria and their differential association with mortality. J Crit Care. (2018) 46:29–36. doi: 10.1016/j.jcrc.2018.04.005 29660669

[39] Proudman CJ Dugdale AH Senior JM Edwards GB Smith JE Leuwer ML . Pre-operative and anaesthesia-related risk factors for mortality in equine colic cases. Vet J. (2006) 171:89–97. doi: 10.1016/j.tvjl.2004.09.005, 16427585

[40] Mair TS Smith LJ. Survival and complication rates in 300 horses undergoing surgical treatment of colic. Part 1: short-term survival following a single laparotomy. Equine Vet J. (2005) 37:296–302. doi: 10.2746/0425164054529409, 16028616

[41] Wormstrand BH Ihler CF Diesen R Krontveit RI. Surgical treatment of equine colic—a retrospective study of 297 surgeries in Norway 2005–2011. Acta Vet Scand. (2014) 56:38. doi: 10.1186/1751-0147-56-38, 24934123 PMC4077634

[42] Cohen ND Woods AM. Characteristics and risk factors for failure of horses with acute diarrhea to survive: 122 cases (1990–1996). J Am Vet Med Assoc. (1999) 214:382–90. doi: 10.2460/javma.1999.214.03.382, 10023402

